# Racial and Ethnic Disparities in High-Quality Home Health Use Among Persons With Dementia

**DOI:** 10.1093/geroni/igaa057.2305

**Published:** 2020-12-16

**Authors:** Shekinah Fashaw, Kali Thomas

**Affiliations:** Brown University, Providence, Rhode Island, United States

## Abstract

Prior research suggests minorities and racially-diverse neighborhoods have decreased access to high-quality hospitals, physicians, and nursing homes. It is not clear how this varies for persons with dementia (PWD) and home health agencies (HHAs). With the Medicare enrollment file, linked to the home health OASIS, the American Community Survey, and Home Health Compare, we examine the influence of individual’s race/ethnicity, as well as the racial/ethnic composition of neighborhoods, on the likelihood of high quality HHA use among PWD in 2016. Minority PWD receiving home health are significantly less likely to use high-quality HHAs than their white counterparts (33% vs 39%, respectively). PWD using HHA in predominantly minority neighborhoods are less likely to use high-quality HHAs compared to PWD in predominantly white neighborhoods (31% vs 40%, respectively). This study is the first to examine racial disparities in the use of HH for PWD. Policy and practice implications will be discussed.

